# Supplementary material for: Analysis of PPARγ Signaling Activity in Psoriasis
Source: Int J Mol Sci. 2021 Aug 10;22(16):8603. doi: 10.3390/ijms22168603 (PMC8395241; doi:10.3390/ijms22168603)
Supplement: Supplementary file 1 [file ijms-22-08603-s001.zip › Supplemental materials_Analysis of PPARg signaling activity in psoriasis/Pathway models/Models images and html files/Interleukin-17 and interleukin-22 signaling in psoriasis/4772.html]

GAB1


# Protein GAB1

|  |  |
| --- | --- |
| URN | urn:agi-llid:2549 |
| Total Entities | 0 |
| Connectivity | 945 |
| Name | GAB1 |
| Description | GRB2 associated binding protein 1 |
| Notes | The protein encoded by this gene is a member of the IRS1-like multisubstrate docking protein family. It is an important mediator of branching tubulogenesis and plays a central role in cellular growth response, transformation and apoptosis. Two transcript variants encoding different isoforms have been found for this gene. [provided by RefSeq, Aug 2008] |

---

|  |  |
| --- | --- |
| Pathway | Prolactin Production |
|  | Nociception Expression Targets Signaling |
|  | NTSR1 Expression Targets |
|  | DRD2 Expression Targets |
|  | DRD2 -> TRPC1 Transcription |
|  | NTRK1/2/3 -> Acetylcholine Production |
|  | Endothelial Cell Dysfunction in Arterial Hypertension |
|  | Smooth Muscle Cell Dysfunction in Pulmonary Hypertension |
|  | IL22 Induces Keratinocyte Proliferation in Psoriasis |
|  | Insulin Resistance in Hepatocytes |
|  | Prolactinoma Overiew |
|  | Hodgkin Lymphoma |
|  | Hepatocellular Carcinoma |
|  | Growth Factor Signaling in Hepatocellular Carcinoma |
|  | Pancreatic Ductal Carcinoma |
|  | IL6 Promotes Inflammation in Muscular Dystrophies |
|  | Medulloblastoma |
|  | Growth Factor Signaling in Neuroblastoma |
|  | Neuroblastoma |
|  | Endometrial Cancer |
|  | Endometrioid Endometrial Cancer |
|  | Clear Cell Ovarian Carcinoma |
|  | High-Grade Serous Ovarian Carcinoma |
|  | Ovarian Cancer |
|  | EGF/cyclinD1 Signaling in Acquired Middle Ear Cholesteatoma |
|  | Subepithelial Fibroblasts in Middle Ear Cholesteatoma |
|  | Cholesteatoma |
|  | HPV E5 Regulates EDNRA and EGFR Signaling Pathway in Keratinocytes |
|  | Steroids Induced Cataract |
|  | Neurotrophic Factor Deprivation in Retinal Ganglion Cell Death |
|  | Cytokines Trigger Otitis Media |
|  | AREG -> NFATC Expression Target |
|  | AREG -> CREB/CREBBP Expression Targets |
|  | AREG -> NCOR2 Expression Targets |
|  | AREG -> HIF1A Expression Targets |
|  | AREG -> AP-1 Expression Targets |
|  | AREG -> FOXO3A Expression Target |
|  | Dopamine/Gi Expression Targets |
|  | BTC -> NFATC Expression Targets |
|  | BTC -> AP-1/ATF/CREB Expression Targets |
|  | Tamoxifen Induced Endometrial Cancer |
|  | EGF -> HIF1A Expression Targets |
|  | EGF -> AP-1/ATF Expression Targets |
|  | EGF -> CREB/CREBBP/ELK/SRF/MYC Expression Targets |
|  | EGF -> MEF/MYOD/NFATC Expression Targets |
|  | EGF -> FOXO3A Expression Targets |
|  | EGF -> TP53 Expression Targets |
|  | EGF -> NCOR2 Expression Target |
|  | EGFR -> NCOR2 Signaling |
|  | EGFR/ERBB3 -> MEF/MYOD/NFATC/MYOG Signaling |
|  | EGFR/ERBB2 -> TP53 Signaling |
|  | EGFR/ERBB2 -> HIF1A Signaling |
|  | EGFR -> AP-1/ATF2 Signaling |
|  | EGFR -> AP-1/CREB/ELK/SRF/MYC Signaling |
|  | EREG -> CREB Expression Target |
|  | EREG -> FOXO3A Expression Target |
|  | EREG -> HIF1A Expression Target |
|  | EREG -> AP-1/ATF Expression Targets |
|  | F2 -> AP-1/CREB/ELK/SRF/SP1 Expression Targets |
|  | F2 -> STAT1/NF-kB Expression Targets |
|  | PLG -> AP-1/CREB/ELK/SRF/SP1 Expression Targets |
|  | PLG -> STAT1/NF-kB Expression Targets |
|  | HBEGF -> FOXO3A Expression Target |
|  | HBEGF -> AP-1/ATF Expression Targets |
|  | HBEGF -> TP53 Expression Targets |
|  | HBEGF -> HIF1A Expression Targets |
|  | HBEGF -> MEF/MYOD Expression Target |
|  | HBEGF -> CREB/MYC Expression Targets |
|  | Receptors and Adaptor Proteins Activated in Cancer |
|  | Proteins with Altered Expression in Cancer-Associated Sustaining of Proliferative Signaling |
|  | Genes with Mutations in Cancer-Associated Sustaining of Proliferative Signaling |
|  | VEGF Independent Angiogenesis in Cancer |
|  | Hyaluronic Acid, CD44 and HMMR in Cancer Cell Invasion and Survival |
|  | LPA Expression Targets |
|  | NRG1 -> MEF/MYOD Expression Targets |
|  | NRG1 -> CREB/CREBBP/ELK/SRF/MYC Expression Targets |
|  | NRG1 -> FOXO3A Expression Targets |
|  | NRG1 -> TP53 Expression Target |
|  | NRG1 -> AP-1/ATF Expression Targets |
|  | NRG1 -> HIF1A Expression Target |
|  | NGF -> SMAD3/NF-kB Expression Targets |
|  | NGF -> AP-1/TP53/MYC Expression Targets |
|  | NGF -> FOXO/MYCN/ELK-SRF Expression Targets |
|  | NGF -> CREB/CEBPB/MEF2A Expression Targets |
|  | AVP/Gs -> CREB/ELK/SRF/AP-1/EGR Expression Targets |
|  | NTS Expression Targets |
|  | AVP/Gs -> MEF/MYOD/NFATC/MYOG Expression Targets |
|  | AVP/Gq -> MEF/MYOD/NFATC/MYOG Expression Targets |
|  | AVP/Gq -> CREB/ELK/SRF/AP-1/EGR Expression Targets |
|  | NTRK -> FOXO/MYCN Signaling |
|  | NTRK -> AP-1/CREB/ELK/SRF/MYC/SMAD3/TP53 Signaling |
|  | FOXO1 Signaling |
|  | PI3K/RAC1 Signaling |
|  | ERK5/MAPK7 Signaling |
|  | ERK/MAPK Canonical Signaling |
|  | TGFA -> FOXO3A Expression Targets |
|  | TGFA -> TP53 Expression Targets |
|  | TGFA -> HIF1A Expression Targets |
|  | TGFA -> MEF/MYOD/NFATC Expression Targets |
|  | TGFA/AP-1/ATF Expression Targets |
|  | TGFA -> CREB/CREBBP/ELK-SRF/MYC Expression Targets |
|  | 2\_Interleukin-17 and interleukin-22 signaling in psoriasis |
|  | 3\_Mesangial cell dysfunction in glomerulonephritis |
|  | 3\_Role of neurotrophic factor deprivation in retinal ganglion cell death |
|  | 4\_2\_Airway smooth muscle cells proliferation and airway tissue remodeling |
|  | Interleukin-17 and interleukin-22 signaling in psoriasis |

---

|  |  |
| --- | --- |
| Group | Genes with Mutations in GWA Studies of Asthma |

---

|  |  |
| --- | --- |
| MedScan ID | 2549 |

---

|  |  |
| --- | --- |
| LocusLink ID | 2549 |
|  | 14388 |
|  | 361388 |

---

|  |  |
| --- | --- |
| Alias | Gab1 |
|  | GRB2-associated binder 1 |
|  | growth factor receptor bound protein 2-associated protein 1 |
|  | GRB2-associated binding protein 1 |
|  | GRB2-associated binding protein 1 long isoform |
|  | AA408973 |
|  | growth factor receptor bound protein 2-associated protein I |
|  | AW107238 |
|  | GRB2-associated binding protein I |
|  | Gab I |
|  | DFNB26 |
|  | GRB2-associated-binding protein 1 |

---

|  |  |
| --- | --- |
| GO ID | 0005070 |
|  | 0038128 |
|  | 0031532 |
|  | 0001525 |
|  | 0007411 |
|  | 0090668 |
|  | 0007173 |
|  | 0008286 |
|  | 0045766 |
|  | 0038089 |
|  | 0014068 |
|  | 0051897 |
|  | 0035728 |
|  | 0038084 |
|  | 0005911 |
|  | 0005829 |
|  | 0007257 |
|  | 0000187 |
|  | 0035924 |
|  | 0008544 |
|  | 0007507 |
|  | 0070102 |
|  | 0060711 |
|  | 0048008 |
|  | 0043410 |
|  | 0030334 |
|  | 0006979 |
|  | 0007165 |
|  | 0005737 |
|  | 0016303 |
|  | 0046934 |
|  | 0004871 |
|  | 0008283 |
|  | 0046854 |
|  | 0036092 |
|  | 0048015 |
|  | 0014066 |
|  | 0038095 |
|  | 0008543 |
|  | 0045087 |
|  | 0048011 |

---

|  |  |
| --- | --- |
| KEGG ID | hsa:2549 |
|  | mmu:14388 |

---

|  |  |
| --- | --- |
| Organism | Homo sapiens {Organism urn:agi-taxid:9606} |
|  | Mus musculus {Organism urn:agi-taxid:10090} |
|  | Rattus norvegicus {Organism urn:agi-taxid:10116} |
|  | Homo sapiens |
|  | Mus musculus |
|  | Rattus norvegicus |

---

|  |  |
| --- | --- |
| Mouse chromosome position | 8 |

---

|  |  |
| --- | --- |
| OMIM ID | 604439 |
|  | 605428 |

---

|  |  |
| --- | --- |
| Rat chromosome position | 19q11 |

---

|  |  |
| --- | --- |
| Hugo ID | 4066 |
|  | HGNC:4066 |

---

|  |  |
| --- | --- |
| Human chromosome position | 4q31.21 |

---

|  |  |
| --- | --- |
| Swiss-Prot Accession | Q13480 |
|  | Q9HA84 |
|  | Q13480.2 |
|  | A0A1B0GS41 |
|  | Q505A4 |
|  | Q9QYY0 |
|  | Q9QYY0.2 |
|  | A8K152 |
|  | Q4W5G2 |
|  | Q6P1W2 |
|  | Q91VW7 |

---

|  |  |
| --- | --- |
| PIR ID | S68442 |

---

|  |  |
| --- | --- |
| GenBank ID | NC\_000004 |
|  | NM\_207123 |
|  | NP\_997006 |
|  | NM\_002039 |
|  | NP\_002030 |
|  | XM\_017007969 |
|  | XP\_016863458 |
|  | XM\_017007970 |
|  | XP\_016863459 |
|  | XM\_017007971 |
|  | XP\_016863460 |
|  | XM\_006714168 |
|  | XP\_006714231 |
|  | XM\_006714167 |
|  | XP\_006714230 |
|  | XM\_017007966 |
|  | XP\_016863455 |
|  | XM\_017007968 |
|  | XP\_016863457 |
|  | XM\_017007967 |
|  | XP\_016863456 |
|  | AC097658 |
|  | AC104685 |
|  | AAY40964 |
|  | CH471056 |
|  | EAX05072 |
|  | EAX05073 |
|  | DQ021880 |
|  | AAY26398 |
|  | JA780910 |
|  | CCH22599 |
|  | JA780912 |
|  | CCH22600 |
|  | AK022142 |
|  | BAB13970 |
|  | AK074381 |
|  | BAB85065 |
|  | AK289767 |
|  | BAF82456 |
|  | AK295684 |
|  | BAH12155 |
|  | AK314635 |
|  | AL049449 |
|  | BC030115 |
|  | BC030751 |
|  | BC064848 |
|  | AAH64848 |
|  | DA109618 |
|  | DA249555 |
|  | U43885 |
|  | AAC50380 |
|  | Q13480 |
|  | NC\_000074 |
|  | NM\_001301298 |
|  | NP\_001288227 |
|  | XM\_006530677 |
|  | XP\_006530740 |
|  | XM\_006530676 |
|  | XP\_006530739 |
|  | XM\_017312576 |
|  | XP\_017168065 |
|  | XM\_011248309 |
|  | XP\_011246611 |
|  | XM\_011248310 |
|  | XP\_011246612 |
|  | XM\_006530674 |
|  | XP\_006530737 |
|  | XM\_017312575 |
|  | XP\_017168064 |
|  | NM\_021356 |
|  | NP\_067331 |
|  | AC116800 |
|  | AC132606 |
|  | CH466525 |
|  | EDL10884 |
|  | EDL10885 |
|  | HM571518 |
|  | HM571519 |
|  | HM571520 |
|  | HM571521 |
|  | HM571522 |
|  | HM571523 |
|  | HM571524 |
|  | HM571525 |
|  | HM571526 |
|  | HM571527 |
|  | HM571528 |
|  | HM571529 |
|  | HM571530 |
|  | HM571531 |
|  | HM571532 |
|  | HM571533 |
|  | AJ250669 |
|  | CAB59832 |
|  | AK045352 |
|  | AK050935 |
|  | AK132176 |
|  | AK155423 |
|  | BC007483 |
|  | AAH07483 |
|  | BC094659 |
|  | AAH94659 |
|  | BX523462 |
|  | CT010264 |
|  | CAJ18472 |
|  | Q9QYY0 |
|  | NC\_005118 |
|  | NM\_001108444 |
|  | NP\_001101914 |
|  | AC\_000087 |
|  | AABR07043601 |
|  | AAHX01097889 |
|  | AAHX01097890 |
|  | AAHX01097891 |
|  | AAHX01097892 |
|  | AAHX01097893 |
|  | AAHX01097894 |
|  | CH473972 |
|  | EDL92297 |
|  | EDL92298 |
|  | GQ140625 |
|  | ACS34829 |
|  | XM\_005262881 |
|  | XP\_005262938 |
|  | XM\_005262882 |
|  | XP\_005262939 |
|  | NC\_018915 |
|  | AMYH02010234 |
|  | AMYH02010235 |
|  | XM\_011248308 |
|  | XP\_011246610 |
|  | AC\_000030 |
|  | AAHY01072438 |
|  | AAHY01072439 |
|  | AAHY01072440 |
|  | AAHY01072441 |
|  | AAHY01072442 |
|  | AAHY01072443 |
|  | AAHY01072444 |
|  | AAHY01072445 |
|  | AAHY01072446 |
|  | AC\_000136 |
|  | ABBA01070001 |
|  | ABBA01070002 |
|  | ABBA01070003 |
|  | XM\_006530675 |
|  | XP\_006530738 |

---

|  |  |
| --- | --- |
| Swiss-Prot ID | GAB1\_HUMAN |
|  | GAB1\_MOUSE |

---

|  |  |
| --- | --- |
| Ensembl ID | ENSG00000109458 |
|  | ENSP00000262994.4 |
|  | ENST00000262994.8 |
|  | ENSP00000262995.4 |
|  | ENST00000262995.8 |
|  | ENSP00000424554.1 |
|  | ENST00000505913.5 |
|  | ENSMUSG00000031714 |
|  | ENSMUSP00000147784.1 |
|  | ENSMUST00000210676.1 |
|  | ENSMUSP00000034150.8 |
|  | ENSMUST00000034150.9 |
|  | ENSRNOG00000017879 |
|  | ENSRNOP00000024390.3 |
|  | ENSRNOT00000024390.5 |
|  | ENSP00000262994 |
|  | ENST00000262994 |
|  | ENSP00000262995 |
|  | ENST00000262995 |
|  | ENSP00000424554 |
|  | ENST00000505913 |
|  | ENSMUSP00000034150 |
|  | ENSMUST00000034150 |
|  | ENSRNOP00000024390 |
|  | ENSRNOT00000024390 |

---

|  |  |
| --- | --- |
| MGI ID | MGI:108088 |

---

|  |  |
| --- | --- |
| RGD ID | 1311085 |

---

|  |  |
| --- | --- |
| Unigene ID | Hs.80720 |
|  | Hs.618456 |
|  | Mm.277409 |
|  | Rn.1725 |

---

|  |  |
| --- | --- |
| Homologene ID | 1542 |

---
